# Stable Lord Madreporic Femoral Stem 32 Years After Revision Total Hip Arthroplasty: A Case Report

**DOI:** 10.7759/cureus.110531

**Published:** 2026-06-09

**Authors:** José Rodrigues, João C Mendes, João Bessa, Pedro Farinha, Maria Parreira, Daniela Isidoro, António Mendonça, Fernando Judas

**Affiliations:** 1 Orthopedics and Traumatology, Coimbra Local Health Unit, Coimbra, PRT; 2 Orthopedics and Traumatology, Unidade Local de Saúde de Coimbra, Coimbra, PRT

**Keywords:** cold welding, long-term outcome, lord femoral stem, lord total hip prosthesis, revision hip arthroplasty

## Abstract

The Lord total hip prosthesis was discontinued because of high rates of failure of the threaded acetabular component and marked proximal femoral bone atrophy related to stress shielding. We report an exceptionally long-term follow-up of a Lord femoral stem that remains stable 32 years after implantation.

In 1994, a 37-year-old tailor underwent left revision total hip arthroplasty (THA) using the Lord system. Fifteen years later, in 2009, he presented in our orthopedic department with pain and substantial functional impairment. Imaging demonstrated aseptic loosening of the Lord acetabular cup with massive bone loss, and revision arthroplasty was performed. Periprosthetic bone loss was reconstructed with 120 cc of impacted cryopreserved morselized cancellous allograft, followed by implantation of a Burch-Schneider (Zimmer Biomet, IN, USA) anti-protrusio cage. The Lord femoral stem showed excellent osseointegration and mechanical stability and was therefore retained. An attempt to exchange the femoral head was unsuccessful due to cold welding at the Morse taper. In 2014, five years after the Lord acetabular revision, radiographs demonstrated fracture of the distal flange of the acetabular cage with lateralization of the implant. However, the patient remained asymptomatic and maintained satisfactory joint function, using a 2 cm shoe lift and a cane. A conservative watchful-waiting strategy was adopted.

In 2026, at the 32-year follow-up, the Lord femoral stem continued to demonstrate clinical and radiographic stability, with no subsidence or progressive radiolucent lines besides secondary acetabular reconstruction challenges. To our knowledge, this represents one of the longest documented follow-up periods for a stable Lord femoral stem in the literature.

## Introduction

Total hip arthroplasty (THA) remains one of the most successful orthopedic procedures, providing excellent pain relief, improved hip mobility, and substantial functional recovery. Nevertheless, only a limited number of implant designs withstand very long-term follow-up. Current literature indicates that only 58% of modern THAs remain in situ at 25 years, and reports extending beyond that period are uncommon [1].

The most common causes of failure are aseptic loosening, instability, and periprosthetic joint infection. Each of these failure modes can lead to substantial acetabular bone loss and, in severe cases, pelvic discontinuity. Bone loss may result from mechanical loosening and osteolysis. Osteolysis is often secondary to polyethylene or metallic wear debris, but it may also develop because of corrosion at modular metal junctions in femoral or acetabular components [2,3].

Management of massive acetabular bone loss and pelvic discontinuity in revision hip arthroplasty remains controversial. Available reconstruction strategies include revision cementless cups, cementless Jumbo cups, reinforcement rings, anti-protrusio cages, cup-cage reconstructions, cup-on-cup configurations, metallic augments, bulk acetabular grafting, and impaction grafting, often combined with specialized bearing options such as dual-mobility or constrained components [4,5]. In extreme cases, particularly in frail or non-ambulatory patients, component removal without reconstruction (Girdlestone procedure) may be considered a final salvage option.

The aim of this report is to describe the 32-year outcome of a cementless Lord femoral stem. At the 15-year follow-up, acetabular revision was required because of aseptic loosening of the cementless Lord cup. The acetabular component was removed, the periprosthetic acetabular bone defect was reconstructed using a bone allograft, and a Burch-Schneider (Zimmer Biomet, IN, USA) anti-protrusio cage was implanted, while the stable femoral stem was retained.

## Case presentation

In 1984, a 27-year-old male tailor presented with advanced left hip osteoarthritis secondary to an acetabular fracture sustained in a motor vehicle accident three years before, which had been managed conservatively. The patient subsequently underwent a left THA. In 1994, ten years postoperatively, a revision THA was performed at the same orthopedic department, using a total cementless Lord prosthesis.

**Figure 1:**
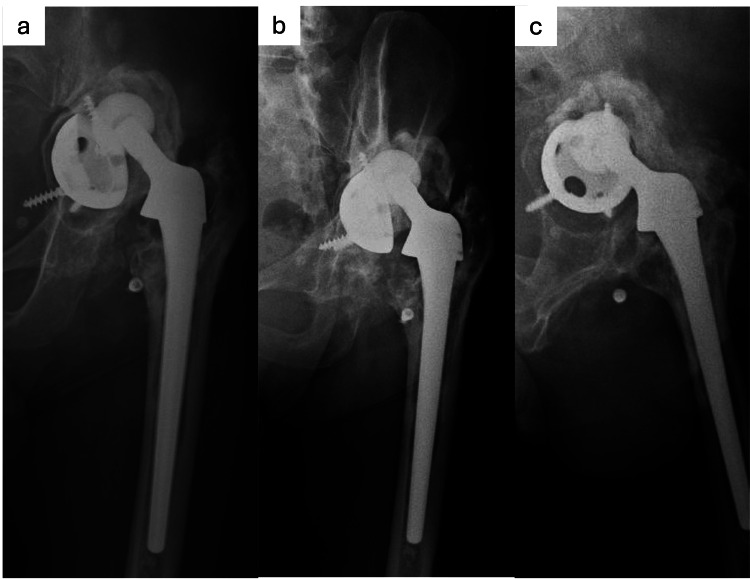
Hip radiographs a, b, c: In different projections obtained at 15-year follow-up after Lord THA (revision total hip arthroplasty), showing aseptic loosening of the acetabular cup with massive acetabular bone loss and stability of the Lord femoral stem.

Fifteen years after revision, in 2009, he was evaluated in our department because of severe groin pain that was refractory to analgesic medication, associated with limb-length discrepancy and a Trendelenburg gait. He required two forearm crutches for ambulation. Radiographic assessment was consistent with aseptic loosening of the acetabular cup (grade III according to Coimbra University Hospitals (HUC) classification) [6], and revision hip arthroplasty was indicated (Figure 1).

**Figure 2:**
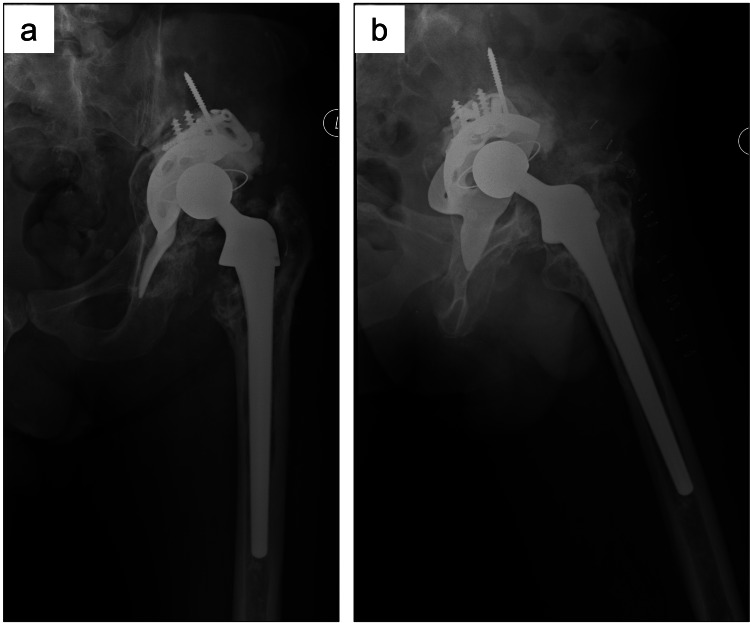
Postoperative hip radiographs a, b: Obtained in 2009 after removal of the Lord loose cup and implantation of a Burch-Schneider (Zimmer Biomet, IN, USA) cage with a cemented polyethylene cup. The massive acetabular bone defect was reconstructed using impaction bone allografting, and the femoral stem was retained.

Using a standard posterior approach, the loose cup was removed, and a Burch-Schneider anti-protrusio cage with a cemented polyethylene cup was implanted. The associated massive acetabular bone defect was reconstructed with impaction bone grafting using 120 cc of morselized cancellous bone allograft provided by the Tissue Bank of the HUC [6]. The Lord femoral stem was stable and therefore retained (Figure 2). An attempt to exchange the femoral head was unsuccessful because of cold welding at the head-neck interface. The perioperative and postoperative courses were uneventful. The patient maintained a slight Trendelenburg gait but was able to walk without external support.

**Figure 3:**
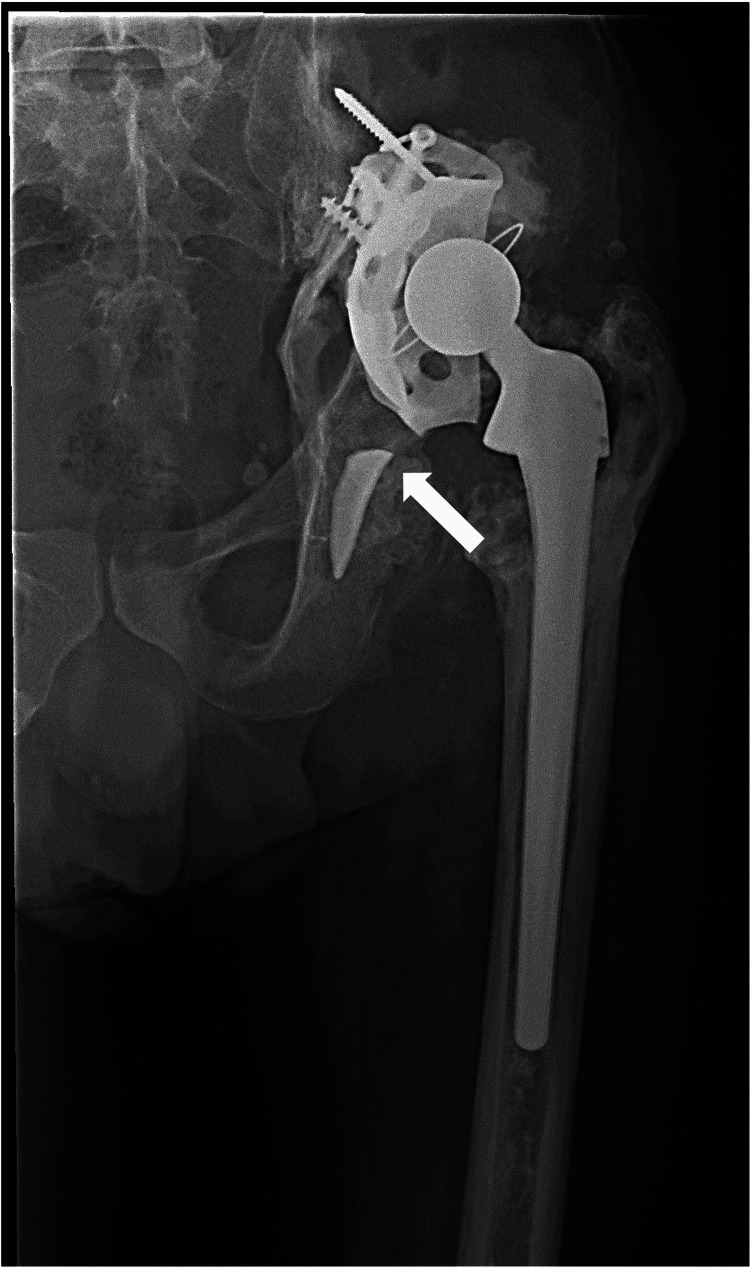
Radiograph findings (2014) In 2014, five years after acetabular revision, imaging showed a fracture of the distal cage flange (white arrow), with lateralization and verticalization of the cage.

In 2014, five years after the second acetabular revision, radiographs showed fracture of the distal flange of the acetabular cage, with lateralization and verticalization of the cage and no evidence of screw failure (Figure 3). Despite these findings, the patient remained asymptomatic, preserved joint function, and continued to have a Trendelenburg-type gait. He was able to continue his professional activity satisfactorily, using a 2 cm shoe lift and a cane.

**Figure 4:**
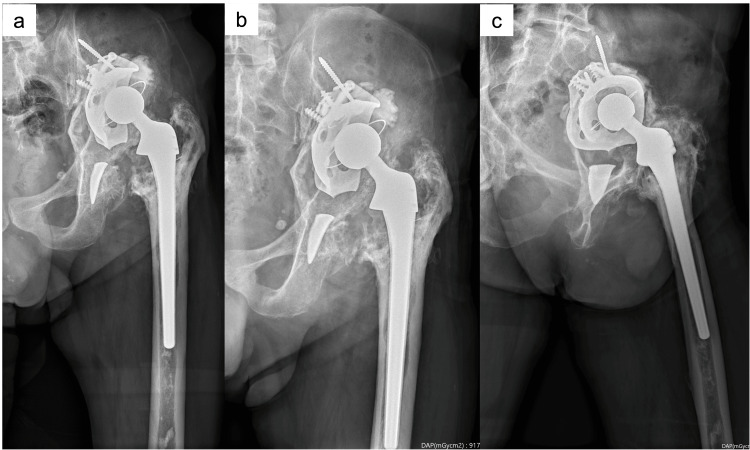
Radiograph findings (2022 and 2026) a: In 2022, eight years after the fracture of the distal flange, imaging showed an acceptable cage inclination angle and stability of the cemented cup. The fixation screws showed no signs of loosening. b, c: In 2026, 32 years after the first total revision hip arthroplasty, the Lord femoral stem remained stable, with no measurable subsidence or progressive radiolucent lines.

In 2022, imaging demonstrated an acceptable cage inclination angle and stability of the cemented cup. The fixation screws showed no signs of loosening, and the femoral stem remained stable without periprosthetic osteolysis (Figure 4).

**Figure 5:**
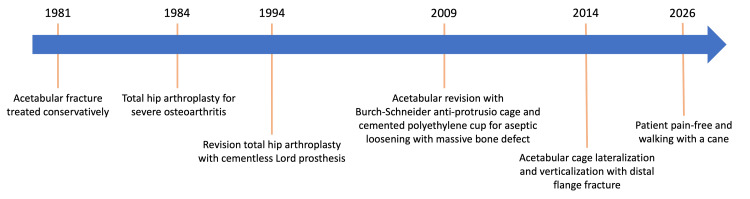
Timeline of the clinical course of the case Burch-Schneider (Zimmer Biomet, IN, USA)

At the most recent follow-up in 2026, 32 years after the first revision THA, the Lord femoral stem remained stable, with no measurable subsidence or progressive radiolucent lines (Figure 4) identified in Gruen zones 1, 7, 8, and 14. No additional cage instability was observed despite the known distal flange fracture. Minimal signs of polyethylene wear were noted, evidenced by slight superior displacement of the femoral head within the acetabular cup. The clinical course is summarized in the timeline below (Figure 5).

## Discussion

Implant longevity remains a major concern in THA despite continuous advances in implant design and surgical techniques. Although the Lord total hip prosthesis has been discontinued, many femoral stems may continue to show long-term mechanical stability after acetabular component revision, as illustrated by the present case.

Use of the Lord THA was abandoned in 1987 at our orthopedic department because of the high failure rate of the cup due to aseptic loosening. Uncoated, smooth-threaded Lord cup provided insufficient contact between the threads and the acetabular bone. Combined with thin polyethylene, poor fixation, and early migration, it led to frequent periprosthetic osteolysis and high revision rates [7].

In the present case, the Lord acetabular cup revision was performed at the 15-year follow-up because of massive periprosthetic osteolysis and aseptic loosening. Bone loss was reconstructed using an impacted cryopreserved morselized cancellous allograft, followed by implantation of a Burch-Schneider anti-protrusio cage with a cemented polyethylene cup. Regarding the cold welding at the head-neck interface, a femoral head that is clinically cold-welded to a stem is one of the most common reasons for unplanned stem removal. The design most often associated with cold welding has been the combination of a titanium stem and a titanium taper [8]. In our case, neither the femoral head nor the stem was replaced.

The Burch-Schneider cage consists of a cup with proximal and distal flanges for fixation to the ilium and ischium, respectively. It is designed to distribute load across the pelvis and bridge areas of insufficient acetabular bone stock. Although acetabular rings and cages provide nonbiologic fixation, they remain cost-effective and still play a limited but valuable role in revision of the most complex cases of acetabular bone loss, including pelvic discontinuity. They can help restore bone stock reliably and provide acceptable survival and satisfactory function at medium- to long-term follow-up [9,10].

Five years after the Lord acetabular revision (2009), fracture of the distal cage flange was documented, accompanied by loss of the initial cage position. However, the cemented cup showed no signs of loosening, the fixation screws showed no evidence of loosening or fracture, and the acetabular reconstruction has maintained surprising mechanical stability over the last 12 years. The patient remains pain-free, without major functional limitation, with a Harris Hip Score of 79.40%, uses a 2-cm shoe lift and a cane, and reports high satisfaction with the surgical outcome.

Repeated flange bending during cage implantation may lead to structural failure. In our case, fracture of the distal flange was probably caused by metal fatigue, possibly related to partial resorption of the bone allograft and subsequent structural failure of the posterior wall. Cage stability depends on the integrity of the posterior wall. Heterotopic ossification, periarticular fibrous tissue, and favorable mechanical positioning of the cemented cup likely contributed to the overall stability of the reconstruction. A conservative watchful-waiting strategy was adopted because of the potential risks of further revision surgery. In contrast, the femoral stem remained stable over time, with no measurable subsidence or radiolucent lines.

In the 1970s and early 1980s, mechanical failure of bone cement was thought to be a major cause of late loosening and periprosthetic bone loss, and cementless THA systems were reintroduced into routine clinical practice. To reduce proximal bone loss, most uncemented femoral stems currently used in primary surgery are only proximally coated. The Lord prosthesis is made of cobalt-chromium alloy and is characterized by a 1-mm beaded porous surface described as madreporic, with a relatively long, fully textured stem. The madreporic surface is created during the casting process. The increased surface area and the crevices created by the beads have been shown to facilitate interlocking once bone ingrowth has occurred [11].

Despite subsidence and proximal femoral bone loss, probably related to greater exposure to polyethylene wear debris and stress shielding, the Lord femoral component has shown good durability and favorable long-term results, with a survival rate of 83% at 21 years when infection is excluded [12,13]. The most obvious disadvantage of this design appears to be the difficulty of removal when indicated because of extensive bone ingrowth, especially along the distal portion of the prosthesis.

## Conclusions

This case demonstrates satisfactory long-term clinical and radiographic success of a complex case at 32 years of follow-up. During the last clinical and radiological examinations, the patient presented an asymptomatic hip and expressed high satisfaction with the surgical result. This outcome may be due to timely surgical intervention by an experienced team and sustained long-term follow-up, evidenced by the successful acetabular reconstruction and the subsequent conservative management of cage fracture without further functional deterioration. Patient-related factors, such as low body mass index and a less physically demanding occupation, may also have contributed to the favorable outcome.

The survival of this THA is remarkable considering the era of implantation and the absence of modern uncemented femoral stems, acetabular cups, porous metals, and highly cross-linked polyethylene. Therefore, the cementless Lord THA remains an important milestone in the history of arthroplasty surgery. To our knowledge, and following review of the literature, this case represents the longest documented follow-up of a stable Lord madreporic femoral stem.
